# Health-related quality of life in abdominal wall hernia: let’s ask patients what matters to them?

**DOI:** 10.1007/s10029-022-02599-6

**Published:** 2022-04-12

**Authors:** O. A. Smith, M. F. Mierzwinski, P. Chitsabesan, S. Chintapatla

**Affiliations:** 1 York Abdominal Wall Unit (YAWU), Department of General Surgery, York & Scarborough Teaching Hospitals NHS Foundation Trust, Wigginton Road, YO31 8HE York, UK; 2 School of Science, Technology and Health, York St. John University, York, YO31 7EX, UK

**Keywords:** Abdominal wall hernia (AWH), Interpretative phenomenological analysis (IPA), Quality of life (QoL), Qualitative, Phenomenology

## Abstract

**Introduction:**

Quality of Life (QoL) is an important consideration in patients with abdominal wall hernia (AWH). What matters to patients and their everyday experience living with AWH may depend on a variety of personal, psychological, social and environmental factors. At present, no study has addressed what is important to this particular group of patients by asking the patients themselves. This study aims to determine QoL from the patient’s perspective by examining the lived experience in this patient population.

**Methods:**

We interviewed 15 patients with AWH until thematic saturation. The patients were purposively sampled from AWH clinic between February 2020 and June 2020 using topic guides and interview schedules. Verbatim interview transcripts were coded and analysed using NVivo12 software and Interpretative Phenomenological Analysis (IPA). We adhered to consolidated criteria for reporting qualitative research (COREQ).

**Results:**

Fifteen participants (8 men and 7 women) of age range 36–85 years, median 65 years, covering all Ventral Hernia Working Group (VHWG) grades. Five superordinate themes were identified each with several subordinate themes, as follows: (1) body image (subthemes—‘changes to perceptions of self’ and ‘fears concerning perceptions of others’). (2) Mental health (subthemes—‘emotional responses’, ‘disruptions to previously solid aspects of identity’, ‘developing coping strategies’). (3) Symptoms (subthemes—‘managing pain’, ‘freedom of movement’, ‘restriction and adaptation of function’). (4) Interpersonal relationships (subthemes—‘difficulties socially connecting’ and ‘changes in sexual relations’). (5) Employment (subthemes—‘financial pressure’, ‘return to work issues’ and ‘costs to family’).

**Conclusion:**

This is the first phenomenological qualitative study in the field of AWH and presents a rich account of what is important to these patients in terms of QoL. Developed from the patients’ own words, the themes are interrelated and should shape our understanding of patients with AWH. This study provides qualitative examples of each theme. This study has identified new themes (body image, interpersonal relationships and employment) that are not incorporated in existing AWH-specific QoL instruments. This is important for surgeons because the study suggests that we are currently not capturing all data relevant to QoL in this specific patient group with current tools. The wider impact of this would be to help counsel patients and support them more holistically through the disease process and it's management. Further research is needed to generate a standardised AWH QoL instrument which incorporates bio-psycho-emotional–social themes important to patients, as identified by patients.

**Supplementary Information:**

The online version contains supplementary material available at 10.1007/s10029-022-02599-6.

## Background

Abdominal wall hernia (AWH) represents a significant and increasing surgical problem [1, 2]. The increasing incidence is due to rising laparotomy rates as well as increasingly older and more obese population with multiple comorbidities [3].

Abdominal wall hernia (AWH) is a chronic disease that can present in one of two ways - as an emergency that requires an immediate life-saving procedure or electively in a planned manner. The preference for both patient and surgeon is the latter [4]. Whilst waiting for an elective procedure, patients need to undergo optimisation, e.g. weight loss management, using a patient-centred pathway as we have described [5]. While awaiting optimisation AWH may progress and affect patients’ Quality of life (QoL).

There is evidence to suggest that AWH patients experience poor QoL [5, 6]. To quote van Ramshorst et al. (2012): “The natural history of abdominal hernias has demonstrated that with time a patient’s quality of life will worsen” [7]. AWHs have significant “physical, social and emotional repercussions” and studies have shown that this results in diminished QoL, as well as issues with mental health and lower satisfaction with body image [5, 6]. Trujillo et al. (2018) established that these patients were “less sexually active, had greater rates of body pain, and had diminished social and physical functioning” [5].

There are many studies that use Health-Related Quality of Life (HRQoL) as an outcome measure for AWH [4, 8–11]. Some studies show that repairing an incisional hernia results in improved HRQoL [12–14]. These studies used different HRQoL tools [15], and furthermore, these tools were generated largely from expert surgeon opinion alone without patient involvement.

Given this, it is difficult to know if these studies captured *meaningful information* pertaining to what is important to AWH patients themselves [6]. We are motivated by The General Medical Council United Kingdom (2020), statement that doctors “should try to find out what matters to patients about their health—their wishes and fears, what activities are important to their quality of life, both personally and professionally—so you can support them to assess the likely impact of the potential outcomes for each option” [16]. Therefore, understanding what matters physically, mentally and emotionally to our patients is of paramount importance.

To understand patient’s QoL, it is critical to explore the lived experiences of patients with AWH and use this information to develop a more holistic approach towards their management. To our knowledge, there are no qualitative studies in this field. The aim of this study is to explore the lived experiences of patients with AWH.

## Methods

### Study design

Phenomenology describes the “total structure of lived experience, including the meanings that these experiences have for the individuals who participate in them” [17]. Interpretative Phenomenological Analysis (IPA) is the qualitative approach to use when exploring these complex human experiences including emotional responses. It aims “to focus on people’s perception of the world in which they live and what this means to them: a focus on people’s lived experience” [18]. This explores human subjective experiences which can be emotionally laden [19, 20].

**Fig. 1:**
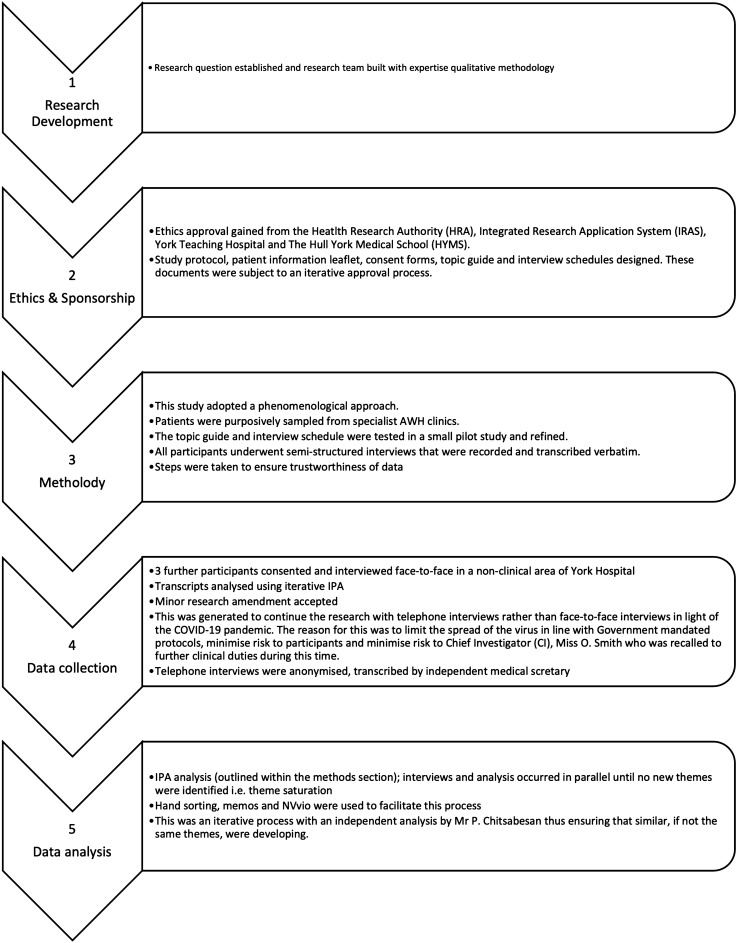
Schematic overview of the steps involved in this study

This study, therefore, uses IPA methodology [21] to examine participants lived experience of a phenomenon, AWH [19]. Our sample size of 15 patients aligns with other phenomenological studies [28–30]. Ethical approval was obtained from The Hull York Medical School, Health and Research Authority, Integrated Research Application System and York & Scarborough Teaching Hospitals NHS Foundation Trust. We adhered to Consolidated Criteria for Reporting Qualitative Research (COREQ) when designing and reporting the study. Figure 1 provides a schematic overview of the study and the steps.

### Participants

We purposively sampled AWH patients with AWH. The patients were identified from a specialist AWH clinic, accessed via authors SC’s and PC’s professional practice. This purposive sample was deliberately, non-randomly selected to produce a diverse range of adult patients (over 18 years old) in terms of demographics and characteristics important in AWH, such as different Ventral Hernia Working Group (VHWG) grades 1–4 [22] and varying complexity [23–25], the presence of comorbidities such as obesity and diabetes, stoma or intestinal fistula; a history of colorectal cancer, ‘benign’ conditions including Inflammatory Bowel Disease and if they were smokers or ex-smokers. We included pre- and post-operative patients, differing socioeconomic and employment status and any ethnicity (although white British is most prevalent in our region). Written consent was obtained prior to interview and verbal consent was audio-recorded.

This sampling technique is used frequently in qualitative research “for the identification and selection of information-rich cases for the most effective use of limited resources” [23, 24]. This initial sample size aligns with other phenomenological studies [25–27].

Patient biographies are outlined in supplementary file 1. These biographies provide context to the lived experiences described below. Pseudonyms were randomly generated for each participant by a member external to the research team.

### Data collection

**Fig. 2:**
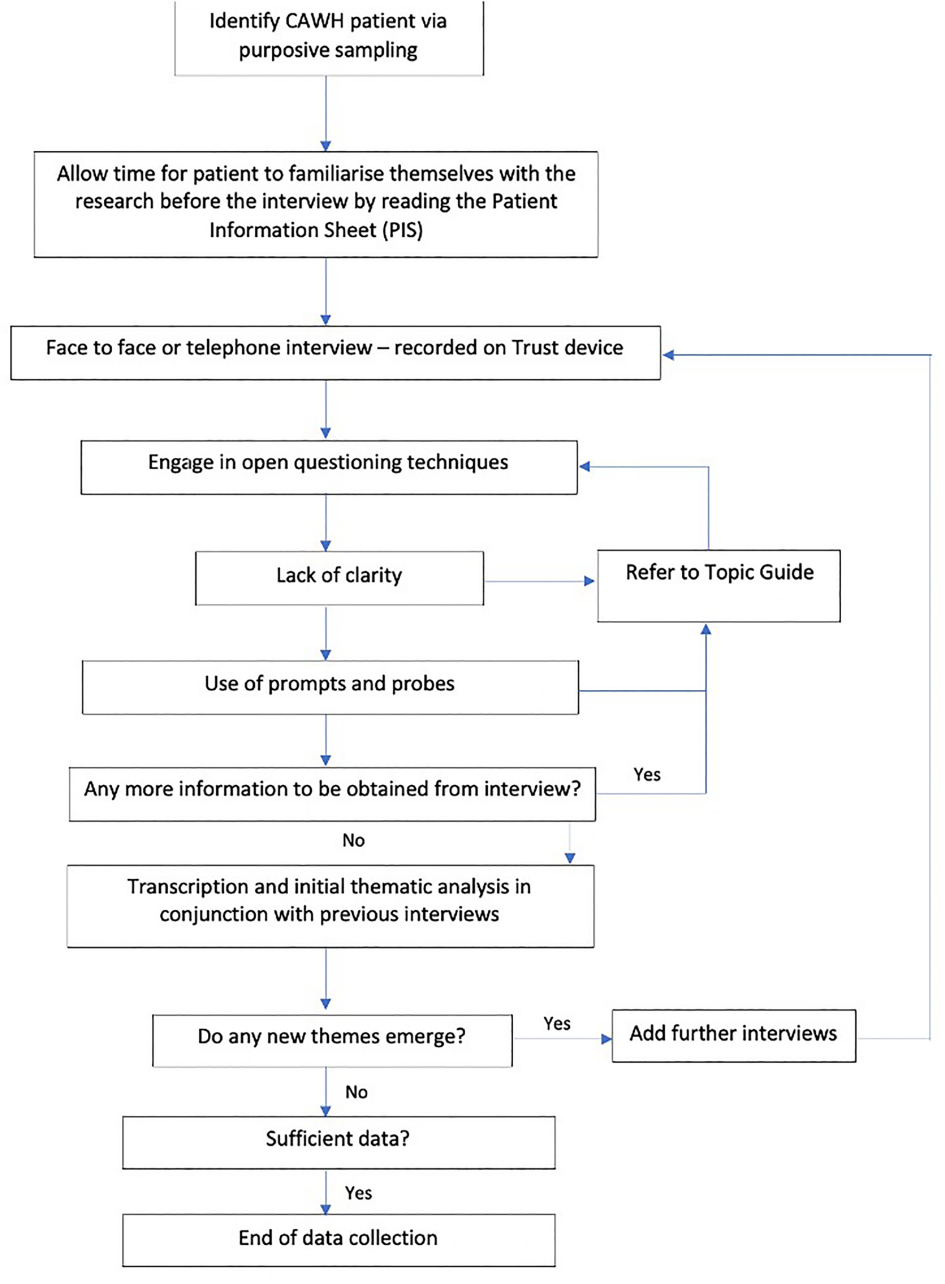
Interview procedure for this phenomenological study. Adapted from: [32]

We developed a topic guide (supplementary file 2) and interview schedule (Fig. 2), which were piloted. These were used to prompt patients during the semi-structured interviews when needed [28] to explore the nature of their hernia, what symptoms they experienced and how this affected their life in a comprehensive manner. Semi-structured interviews were chosen since they draw out the lived experience of a phenomenon, in this case AWH [29]. Each participant took part in an interview with the chief investigator (OS) between February and June 2020. The first three participants were interviewed face-to-face in a non-clinical area in our hospital and the remaining 12 were interviewed via telephone in their own homes due to unexpected pandemic and COVID-19 Government mandated restrictions. These were continued until *thematic saturation*, which is the point at which no new data emerge from further sampling [19, 21, 30, 31]. Interviews lasted between 45 and 90 min and were audio-recorded and stored in accordance with the hospital's information governance policy. They were then transcribed verbatim by OS and an independent medical secretary not involved in the research. These transcripts were compared for any discrepancies or errors.

### Analysis

Data were analysed by chief investigator OS using NVivo v12 [33], a qualitative analysis software that allows coding and analysis of emergent themes with the ability to integrate, change and review codes. As per IPA process protocol, transcripts were read and reread [21]. Analysis was done using IPA methodology and interviews continued until thematic saturation was reached which in this case was the point at which no new super or subordinate themes emerged. This allowed identification of insights as well as careful documentation of descriptive, linguistic and conceptual comments [21]. Emergent themes developed allowing identification of heterogeneity across themes, subsumption (emergent theme itself acquires a super-ordinate status since it converges several themes), polarisation (oppositional relationships), contextualisation and numeration (frequency) [34]. Connections across these themes were identified prior to analysing the next transcript. This was a reiterative process and was repeated until thematic saturation.

The chief investigator engaged in reflexivity (see supplementary file 3) and bracketing practices prior to triangulation and subsequent analysis [34]. Following analysis, of all transcripts, patterns across the themes were carefully examined and superordinate themes were generated. All emergent themes were discussed with authors PC and SC (triangulation) as well as a qualitative researcher and an academic sociologist (MM). PC reviewed and analysed the last three scripts independently to check concordance with the ideas and codes formulated by OS. This was examined and modulated based on discussion and emersion. This approach generated a digitised audit trail thus allowing themes to be traced back to each participant account. Throughout the process, strategies were put in place to ensure study quality, rigour and trustworthiness [34] and are summarised (supplementary file 5, Table 1).

## Results

Fifteen participants took part in this study (8 men and 7 women) with an age range 36–85 years (median 65 years, interquartile range 30 (45–75). Table 1 provides a summary of participant demographics.

**Table 1:** Study participant demographics^1,2^

Participant name	Sex	Age	VHWG grade	CT hernia dimensions craniocaudal length × axial width (cm)	NHS/private	BMI post rehabilitation	Smoker	Diabetic	Previous wound infection	Stoma	Previous cancer	Fistula	Socioeconomic status	Employed	Post op/pre op	Telephone interview
Agnes	F	65	2	15.4 × 12.6	NHS	29.6	Ex-smoker	No	No	No	Yes	No	Middle	Yes	Pre-op	No
Betty	F	63	1	15.7 × 5.2	Private	26.1	Never	No	No	No	No	No	Upper	Retired	Pre-op	No
Charlotte	F	68	4	13.4 × 16.9	NHS	38.6	Ex-smoker	Yes	Yes	No	No	No	Middle	Retired	Pre-op	No
David	M	61	2	13.5 × 5.7	NHS	31.2	Never	Yes	No	No	No	No	Lower	Yes	Pre-op	Yes
Eric	M	78	1	6.5 × 19.1	NHS	29.9	Ex-smoker	No	No	No	No	No	Middle	Retired	Pre-op	Yes
Frank	M	75	4	22.7 × 17.4	NHS	25.8	Ex-smoker	No	Yes	No	Yes	Yes	Middle	Retired	Post-op	Yes
George	M	45	3	13.9 × 12.6	NHS	30.5	Ex-smoker	No	Yes	Yes	No	No	Lower	Yes	Pre-op	Yes
Harry	M	84	2	22.4 × 15.3	NHS	28.8	Ex-smoker	Yes	No	No	Yes	No	Upper	Yes	Post-op	Yes
Ian	M	58	4	7.1 × 6.9	NHS	30.2	Ex-smoker	No	Yes	No	Yes	No	Middle	Yes	Pre-op	Yes
Joan	F	75	3	10 × 9.1	NHS	26.3	Never	No	No	Yes	Yes	No	Middle	Retired	Pre-op	Yes
Kevin	M	74	3	11.6 × 8.2	NHS	32.4	Ex-smoker	No	No	Yes	No	No	Middle	Retired	Post-op	Yes
Lisa	F	39	1	–	NHS	29.2	Never	No	No	No	No	No	Middle	Yes	Post-op	Yes
Marge	F	36	1	–	NHS	20.4	Never	No	No	No	No	No	Middle	Yes	Post-op	Yes
Norman	M	77	3	11.6 × 14.8	NHS	24.1	Ex-smoker	Yes	No	No	Yes	No	Middle	Retired	Post-op	Yes
Ophelia	F	44	1	5.8 × 6.1	NHS	30.7	Never	No	No	No	No	No	Middle	Yes	Post-op	Yes

^1^Names given to participants throughout the thesis are pseudonyms, ensuring anonymity

^2^Key: M = male; F = female; NHS = National Health Service, United Kingdom; VHWG = Ventral Hernia Working Group; BMI = Body Mass Index

Data analysis identified five superordinate themes, each with several subordinate themes. The superordinate themes are:

1. Body image
2. Mental health
3. Symptoms
4. Interpersonal relationships
5. Employment.

**Fig. 3:**
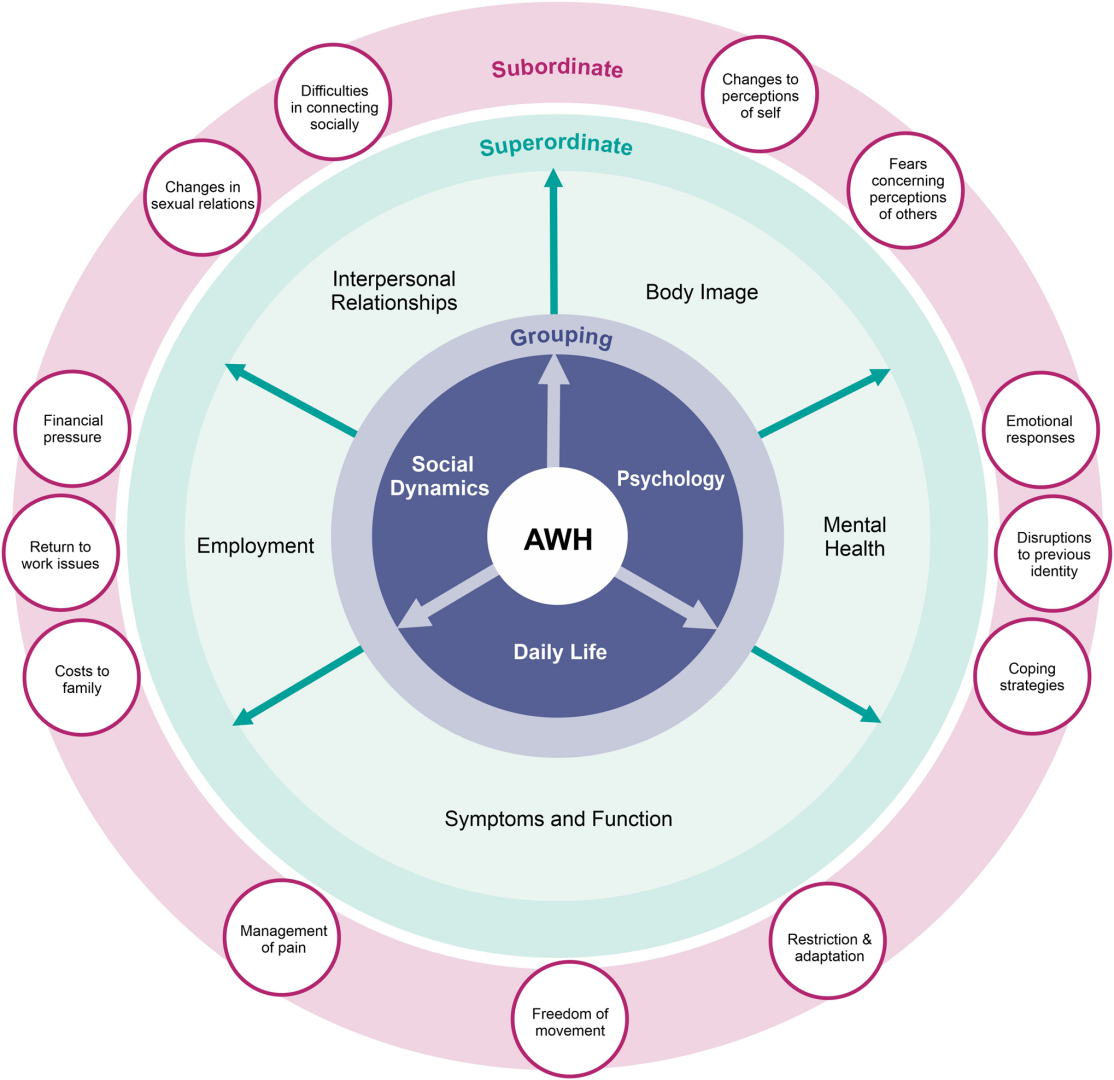
A diagrammatic representation of groupings, superordinate themes and related subordinate themes (York Model of QoL in AWH)

These will be described in greater detail below. Figure 3 provides a diagrammatic representation of groupings, superordinate themes and related subordinate themes.

## Superordinate theme 1: body image

### Subordinate theme: changes to perception of self

All participants mention body image as a salient issue and struggled with self-perception with some using metaphor and imagery to identify themselves as grotesque characters, demonstrated by the following quotes.

“*I think that the biggest issue is that I feel like Mr Blobby. I walk like Mr Blobby or like Jar Jar Binks.” (Betty)*

“*I’ve got that many lumps and bumps now... I’m just like an elephant man.” (George)*

It was difficult to ascertain the extent that these participants really identified with these characters or if these responses were driven by emotional responses to intrusive remedial surgery, or both. Irrespectively, many male and female participants used the imagery of a pregnant woman to capture their profile and how they see themselves. Most male participants found this profile emasculating, whilst females expressed detrimental consequences in terms of distress and loss of control over physical appearance.

### Subordinate theme: fears concerning perceptions of others

A key part of patients’ disrupted body image was informed by their fears of other peoples’ perceptions of them. They felt that other people viewed their abdomens as “*different*” or “*abnormal*” in terms of appearance and function.

“*it felt like a burden I was carrying around, but the main burden not being the pain of the condition, but the weight of what other people were thinking” (Ophelia)*.

Participants often have the impression that others felt uncomfortable around them or were somewhat shocked or disgusted by their body, which led to them actively avoiding social situations.

Occasionally participants’ body image was positively reinforced through social interaction. Typically this occurred post-operatively when there was a stark change in body profile. Participants described family and friends being astonished by the visible difference and comments regarding “*looking well*” or “*physically fitter*”.

## Superordinate theme 2: mental health

### Subordinate theme: emotional responses

AWH had a profound effect on mental health leading to low mood, depression and anxiety. This psychological impact of the hernia itself often occurred against a backdrop of prior surgical complications or, in older participants, co-morbidities. One participant described the restrictive nature of his hernia both physically and mentally, stating “*it just gets to me…it’s like being in a prison cell” (David)*.

This serves as an intriguing metaphor of punitive punishment, captivity and a loss of basic rights. Others worried about recovery, employment and intimate relationships. Whilst pre-operative participants evoked sentiments of frustration, apprehension and dissatisfaction, post-operative participants expressed relief and hopefulness for the future. However this was not unanimous, juxtaposed feelings of apprehension and hope about the future was shared by some.

### Subordinate theme: disruptions to previously solid aspects of identity

All participants reported, to varying degrees, the feeling of identity loss and the difficulty to integrate with and adjust to new identities. For example, that of the “*sick person*”, “*the unemployed*”, “*the false mother*”.

Loss of identity was difficult especially for Marge and Lisa who are young women with careers in the fitness industry who expressed views that they should and are expected to “*look a certain way*”, a way of social expectation that they were unwilling to let go of.

Similarly, for Frank, a large element of his identity rests in being ex-military. He prides himself on being physically fit in his youth and representing his country. He shared a particularly vulnerable experience where he was asked to be a pallbearer at a squadron friend’s funeral. Whilst advised by his surgical team not to lift heavy objects and to wear his Vac-Pac wound dressing he deliberately ignored this advice because he expressed immense feelings of guilt and shame by not doing his perceived duty. The following quote from Frank demonstrates the important role core values play in the sense of self:

“*I actually left it in the car, the Vac-Pac, wore a big overcoat and managed to assist the team with the coffin…for the service. I was very conscious of it and…my role*”.

61-year-old David's identity as a grandfather was disrupted due to no longer being able to actively play with his grandchildren causing him to fear *their memories of him as a *“*sick person*”.

### Subordinate theme: developing coping strategies

The majority of patients acknowledged that their hernia journey was one of slow progressive growth and that it took them time to come to terms with the diagnosis. All participants developed coping mechanisms to manage challenges posed by their AWH, which ranged from psychological, spiritual, physical and social.

Psychological strategies, often previously acquired in their pre-diagnosis life, helped patients deal with daily life. Frank described that he learned to cope by having to “*learn how to ask for help… and had to reprogram my mind…to ask for help sometimes*.” Others noted that their coping strategies changed over time. Spirituality played a role too. In general, this was solitude based rather than socially informed spirituality. Introspective mechanisms, such as mindfulness and self-care routines, were described as important and valuable coping mechanisms. It seemed that one way to deal with a physical impairment or change was to seek greater control over the psychological process.

Some found that engaging in previously physically enjoyed activities, like running, were markers of improvement, success and justification for surgery. Others engaged in newer lifestyle mechanisms to cope. Examples included exercise, preparing healthy meals and engaging with personal passions, all of which reportedly elevated mood, whilst actively seeking to rehabilitate/change previously cited notions of an impaired/sick body.

Social strategies were highly important. Whilst some patients removed themselves from the public sphere, socialising with family, friends and employees in more familiar settings were described as improving self-confidence and helped patients cope. The contrast here could be due to differences between fears of strangers’ perceptions compared with socialising with trusted people who had knowledge of patients’ pre-operative bodies.

## Superordinate theme 3: symptoms

### Subordinate theme: managing pain

Pain was a dominant, negative factor that all patients struggled with. It was linked to psychological distress, loss of identity, decreased quality of life, restricted movement, job loss and loss of intimate relationships. This resulted in a need to redefine a new “normal” for some.

“*I have had a couple of occasions when I woke up in the night literally sweating with the pain, and it was excruciating” (Agnes).*

There is perhaps a difference between medicating and “*dealing with pain” (Norman)* and living with constant pain/discomfort despite medication, the latter having greater psychological affect.

### Subordinate theme: freedom of movement

Hernias can have a great impact upon mobility and participants described issues with balance, bending and restrictions upon their movement. They reported the need to relearn or adapt certain activities of daily living, such as personal care, walking, adapting stance, and adapting bending to pick up objects.

“*I couldn’t be doing things in a normal kind of way. I mean they became the norm, so you know in the end you just deal with it and accept it and that’s the way it is. But to start with it does feel quite frustrating that it’s not right and you just kind of long for when it will be” (Ophelia*).

Young mothers found this particularly distressing since it impacted upon childcare. “*If she wanted a cuddle (daughter) I would sit on the sofa without picking her up… I just adapted. But, it wasn’t very nice” (Lisa)*

These symptoms had a knock-on effect on mental health, employment, self-perception and when presented were viewed by participants as restrictive in their daily lives.

### Subordinate theme: restriction and adaptation of function

All participants described overwhelming frustration at the inability to do things previously enjoyed due to symptoms associated with their hernia. This was depicted with expressions, such as “*trapped*”, “*restricted*”, “*stuck*” and “*imprisoned*”.

“*There was just an awful lot of adaption in terms of what I was physically able to do. A, because I didn’t want to feel like I made it worse whether the hernia would come out, but also the pain” (Lisa)*

Everyday tasks like shopping, getting out of bed, showering and getting dressed were affected. Male and female participants elected not to wear “*enhancing*”, “*figure-hugging*” or “*tight*” clothing and, opted for “*concealing*”, “*baggy*” clothing. Their focus was not one of fashion but rather to cover bulges and to mentally cope with feelings of fatness or ugliness. As Kevin said, “*clothing?! It was just covering up!*”

## Superordinate theme 4: interpersonal relationships

### Subordinate theme: difficulties socially connecting

All patients described issues of loneliness with some describing a feeling of “*social demotion*” due to loss of self-identity.

Discussing problems related to their AWH allowed participants to reclaim a sense of control over their lives by verbalising and exploring solutions. This allowed the emergence of new future possibilities. Betty used the following metaphor, “*it’s a little bit like changing goal posts*.”

In contrast, others like George suffered job loss and depression and appeared to deliberately disconnect. He purposefully distanced himself from others to avoid sympathy, feeling inferior and “*uncomfortable situations that feel stifling*”.

However, *Joan*, became an active member of national hernia and stoma support groups to feel “*less alone*” suggesting that only those who share similar lived experiences can understand them.

### Subordinate theme: changes in sexual relationships

Intimate sexual relationships were negatively affected by AWH. “*You tend not to look at yourself anymore because it really is quite a horrible looking thing and avoid mirrors and my wife’s not allowed to look at me anymore…” (Eric)*

Some participants stopped completely due to pain or feeling vulnerable around their partners.. “*It (sexual intercourse) had virtually stopped” (Harry)*.

George had recently started dating a new partner and “*tends to avoid sexual encounters deliberately*” like others due to feelings of shame, inadequacy and a fear of rejection. Some expressed guilt for not feeling like they wanted to have sex and remorse at discouraging their partner.

## Superordinate theme 5: employment

### Subordinate theme: financial pressure

Financial pressures were an issue, secondary to unpaid sick days, hospital visits and in some circumstances job loss. Some described the direct effect that this has had on mental health and motivation. Others experienced a threat to identity and a struggle to maintain that sense of self. This had a profound effect on these participants to the point where they considered applying for other jobs.

### Subordinate theme: return to work issues

Participants reported two forms of struggle in the workplace. The first relates to difficultly undertaking the physical task required at work, the second involves the psychological struggle of “*letting teammates down”…(Lisa)*.

This coupled with feelings of guilt and disappointment at not being about to pursue their vocation resulted in inner conflict for many and self-deprecation. Others questioned their abilities as fitness instructors, their career choice as well as their role a financial provider within their family units.

### Subordinate theme: costs to family

Both male and female participants who had experienced involuntary job loss or financial difficulties due to employment issues related to their hernia highlighted that it negatively affected their view of themselves as a provider for their families.

“*I unfortunately lost that job because of it because they couldn’t afford to keep me on long time sick….”(Ian)*

Most had not experienced job loss before and were in “professional” careers such as teaching, and they did not consider themselves ready to accept changes in employment initially.

Some participants cultivated different strategies such as helping others through volunteering and others considered downsizing their house to reduce expenditure. Others demonstrated openness to new ways of thinking and to new situations to help with their role as a provider.

Post-operative participants returning to work reported improved self-esteem, improved confidence and decreased psychological distress.

## Discussion

This is the first in-depth study of QoL uniquely using a phenomenological approach in AWH patients by investigating their lived experience. We identified themes in this study that illustrate the complex interactions between significant AWH and psychology, daily life and social dynamics. The superordinate themes are distinctly categorised to aid ease of readability, but the reality is that these themes are interlinked, and it is important to realise that the “lived experience” is not a linear journey. Such experiences are complicated and fluid with an ongoing process of readjustment, changing perceptions of self and perceived challenges. The aim of this study was to provide an overview of the qualitative co-constructed socially informed QoL themes which are important to AWH patients.

This topic may be discussed from sociological and surgical perspectives. From a sociological standpoint, the findings illustrate the significance that patients place upon their external body in terms of both appearance and of it's ability to perform everyday activities and work-based tasks. The patient narratives reveal that body image and the aesthetics associated with this played a significant role in their sense of self, which was often informed by gender, family, professional and occupational identities. Experiencing AWH proved a significant emotional burden for patients and heightened their levels of self-consciousness. As such, some patients expressed arguably irrational or uninformed fears of other peoples’ perceptions of their ‘*grotesque*’ bodies, demonstrating a dominant ‘body-beautiful’ complex that permeates across many Western societies within the twenty-first century [35–38]. Likewise, a notion of ‘body as machines’ was evident in patients’ striving to ‘fully fix’ their perceived ‘broken’ bodies by returning to pre-operative levels of performance, often set through goal setting [39]. Patients used other coping strategies to combat the psychological, emotional and physical distress caused by AWH including psychological training and peer support groups, well documented in the literature to improve psychological resilience [40].

Researchers have recently suggested that factors like body image and employment must be important to AWH patients and likely have been omitted from current AWH QoL tools [15]. Until now, this was anecdotal and was not proven by rich empirical data. Our research team recently critiqued the existing AWH-specific QoL tools [41] and we shall now consider the themes identified by this study in the context of these instruments.

### Study themes in the context of existing hernia AWH HRQoL instruments

The question that must be asked is whether current tools provide meaningful indicators of HRQoL for AWH hernia patients. Six AWH-specific tools were identified via wide literature search (supplementary file 4). These are: the Carolina Comfort Score CCS [8]; the Hernia Related Quality of Life Scale (HerQLes) [42]; the Activities Assessment Scale (AAS) [43]; the Abdominal Hernia-Q (AHQ) [6]; the Hernia-specific Quality-of-Life assessment instrument (HERQL) [44] and the European Registry of Abdominal Wall Hernias QoL Score (EuraHS-QoL) [45].

Please note that the AAS is only included here under the umbrella of CAWH specific tools since it has been outlined as such in a systematic review Grove et al. [15] and by Majeed et al. [46]. Although it  is possible that the authors of these systematic reviews looked at the origins of the tools identified via their search strategies this is not addressed within their papers. Cherla et al. have used the AAS to measure HRQoL in CAWH patients [47]. Otherwise, it has been used as a reliable and valid instrument to evaluate patient function in two different patient populations—laparoscopic and open groin hernia [48] and in women post pelvic reconstruction surgery [43]. It is therefore possible that it has been inappropriately used as a HRQoL tool in the paper by Cherla et al. [47]. We authors of this paper believe that the AAS may more appropriately fall under the title of a “functional assessment tool” and we should not automatically equate function with HRQoL. That is overly simplistic and according to our study findings, would only cover one small aspect of this larger, more complex topic.

These aforementioned HRQoL tools have positive aspects and limitations, which have been outlined elsewhere [15]. Positively, they represent an important step away from a “one size fits all” approach to HRQoL. However, each tool is insufficiently validated and without the appropriate psychometric properties [49]. Beyond this, none of the tools were developed with the involvement of patients from the ‘ground up’ i.e. identification of themes from the patients themselves before questionnaire development [50]. Authors of the AHQ did involve patients in focus groups and had debriefing interviews but they commented on a tool that was already designed by experts [6]. These are distinct patient populations who may hold different opinions pertaining to HRQoL that are not comparable. Ultimately, we cannot assume to know what is important to patients in terms of their QoL without asking them [50].

This view is cemented by supplementary file 4, which provides a summary as to whether the current AWH-specific tools include themes identified by this study. None of the current tools include all the themes identified here and most seem to concentrate on pain and function which may mean they do not provide a holistic examination of AWH patients’ HRQoL (supplementary file 4 provides a summary of the current AWH-specific tools including their themes).

When an existing AWH-specific tool touches upon a theme it often does so incompletely. For example, the CCS asks patients specifically about mesh related pain and does not consider issues with balance, bowel or urinary symptoms. Another example includes the HERQL which asks about ‘economic burden’ associated with AWH however, there is a lack of clarity to what this means i.e. the loss of job or loss of finances due to sick days. Only two of the tools ask about cosmesis and they do so superficially. None consider the effect of AWH on interpersonal relationships. Two tools include items related to mental health — the HerQLes and AHQ. Importantly, they do not explicitly ask about low mood/depression and anxiety related to the AWH itself. Furthermore, no tool includes items related to embarrassment and shame, which have been highlighted as important themes related to QoL in this study. Interestingly, all tools ask about pain. This may be sociologically linked to other research that highlights that as a society we are content to discuss, normalise and manage pain in daily life [51]. Perhaps it also reveals the positivist mind set of the surgical experts that have designed the tools and who are likely more symptom centric. Questions relating to sexual activity and the impact upon sexual relationships are also sparse. This may be in part due to cultural reticence to divulge and “discuss such personal matters” [52].

### What are the implications for clinical practice?

This study helps us understand how AWH can impact on HRQoL and that this is a complex mix of physical, psychological, emotional and social issues that are pertinent to all patients but affect each of them in different ways.

We propose that this understanding is essential for surgeons who manage significant AWH, and perform Complex Abdominal Wall Reconstruction. With current good practice guidelines [16], promoting a patient-centred approach to ensure patients are aware of the individualised risks and benefits of an intervention, it is critical that surgeons understand how a patient’s hernia affects their QoL. This would allow them to provide realistic expectations and appropriate counselling regarding what surgery could realistically achieve. A shared decision can be reached that both patient and surgeon have contributed  to fully. There are new guidelines on optimal management for patients with rectus diastasis with concomitant umbilical hernias [53] and we believe these QoL issues may also be relevant in the discussions with the patient.

Being cognisant of this, and by acknowledging the themes identified in this study increases an AWH surgeon's awareness of QoL in general and it may help surgeons to “not only add years to life but also life to years” [54]. We humbly propose that understanding HRQoL from the patient’s perspective is crucial to managing all patients and teaching this understanding, as well as incorporating these concepts in everyday practice, should be instilled from medical school.

We also believe that focussing on the patients’ voice helps us identify what domains we need to centre on in QoL research. Increasingly we note that grant applications require us to consider how a surgical intervention is perceived in terms of patient’s quality of life.

The aim here has not been to say how HRQoL should be measured but it is clear that none of these tools are holistic. This does not mean that the tools themselves are completely insufficient or lack utility in clinical practice, but they do not include information that patients have identified as being important to themselves in this study. Therefore, this work could be sensibly used to inform future AWH HRQoL tools or revisions of existing tools.

### Limitations

The majority of interviews were telephone interviews due to the COVID-19 pandemic. This means that subtle nuances of body language will have been missed, which may have allowed the interview to evolve in a different direction, but, it could be argued that using this platform aided discussions surrounding sensitive issues, adding to the quality of the data.

The aim of an explorative qualitative study is to obtain a breadth of the subjective views across a narrative, and researchers can often use mixed methods and data gathering tools to ensure they reach thematic saturation [55–57]. This is not seen as a criticism of qualitative research but as a strength that quantitative methods often cannot utilise.

Also, the credibility and the trustworthiness of the face-to-face and telephone interview data are valid for exploratory qualitative purposes. The accuracy of face-to-face interviews and phone interviews was not compromised as all interviews were recorded and transcribed verbatim, whilst the same interview guide was used for all participants. The interview guide had been developed during face-to-face preparatory interviews and hence provided a strong foundation to build on.

This study was conducted in the United Kingdom where all participants were British nationals and this may bear some importance on generalisability of the results. Culture, language and nationality will undoubtedly have some influence on the lived experience of AWH and so it is possible the results of this study may not be generalisable to different populations and different health care systems; however, with such emotive themes, it is likely there will be some shared aspects.

## Conclusion

This is the first qualitative study in the field of AWH and presents a rich account of what is important to these patients in terms of their HRQoL. Developing from the patients’ own words, the themes presented are interrelated, should shape our understanding of patients with AWH and provide versimilitude to the many discussions on their QoL. It is abundantly clear that our current understanding of HRQoL is lacking, especially in the field of AWH. This study has identified new themes that are not incorporated in existing AWH-specific HRQoL instruments. This demonstrates that, if as a society, we want to understand how disease processes affect QoL and how interventions can change this, it would need to invest more time and effort into both identifying what makes up QoL for patients and how it is possible to investigate such a complex non-linear interrelated set of issues. The wider impact of this would be to help counsel patients and support them more holistically through the disease process and it's management. Moving forward, this may allow for more actionable intervention and patient-centred care such as at our hospital where we have created a York process pathway for these patients with AWH that aims to manage multiple issues [58].

## Supplementary Information

Below is the link to the electronic supplementary material.

Supplementary file1 (DOCX 25 KB)

Supplementary file2 (DOCX 22 KB)

Supplementary file3 (DOCX 17 KB)

Supplementary file4 (DOCX 20 KB)

Supplementary file5 (DOCX 24 KB)

## Data Availability

Not applicable.
