# Supplementary material for: Health-related quality of life in abdominal wall hernia: let’s ask patients what matters to them?
Source: Hernia. 2022 Apr 12;26(3):795–808. doi: 10.1007/s10029-022-02599-6 (PMC9003180; doi:10.1007/s10029-022-02599-6)
Supplement: Supplementary file 3 — Supplementary file3 (DOCX 17 KB) [file 10029_2022_2599_MOESM3_ESM.docx]

**Supplementary file 3:** Chief investigator (author OS) reflexivity statement

Bracketing is an essential part of descriptive phenomenology. It is also referred to as phenomenological reduction and is a process whereby the researcher must adopt a phenomenological attitude and bracket their pre-conceptions. By engaging in bracketing, I reflected upon and identified preconceived ideas related to the phenomenon thereby allowing the research to view experiences from the participants perspective.

It is well known that observing/measuring a phenomenon has the potential effect of changing that phenomenon – “observer bias”. This is unavoidable to some degree since researchers are rarely completely neutral regarding their research. To avoid this, I have engaged in reflexivity. I have an awareness of my place in the socio-cultural context and reflecting upon this will prevent me from reinforcing harmful biases. I am aware that my social position as a cis-presenting, Caucasian, young, female doctor with high educational achievements, CAWH operative experience, surgical and research training may affect how patients relate to me and equally, might influence how I interpret data. It may result in my making assumptions and noticing certain aspects of a phenomenon described whilst being blind to others. These elements may be viewed both negatively and positively. Positively, having research and clinical training will keep me focused on the application of quality research, within time constraints. Negatively, my previous research experience has largely been quantitative and, doctors tend to be very goal-orientated and systems based, which means I need to be aware of this and focus more on the stories these participants are recounting, interpret these and defend them. Whilst I do hold quite positivist views concerning aspects of research, I equally hold a relativist standpoint and believe that certain views are relative to differences in perceptions. Reflecting upon this through a qualitative journal, peer debriefing and by critically examining myself as well as my views through a professional and personal lens shows self-awareness that is vital in qualitative research.
