# Supplementary material for: Health-related quality of life in abdominal wall hernia: let’s ask patients what matters to them?
Source: Hernia. 2022 Apr 12;26(3):795–808. doi: 10.1007/s10029-022-02599-6 (PMC9003180; doi:10.1007/s10029-022-02599-6)
Supplement: Supplementary file 4 — Supplementary file4 (DOCX 20 KB) [file 10029_2022_2599_MOESM4_ESM.docx]

**Supplemenary file 4** – Summary of CAWH specific HRQoL tools and whether they include themes identified by this study^1^

|  | **Is the theme considered in the CAWH HRQoL Tool?** | | | |  |  |
| --- | --- | --- | --- | --- | --- | --- |
| **Theme identified in this study** | **AAS** | **CCS** | **HerQLes** | **EuraHS-QoL** | **HERQL** | **AHQ** |
| **Year** | 1995 but published in 2005 | 2008 | 2012 | 2012 | 2017 (for inguinal hernia)  2020 (for CAWH) | 2020 |
| **Country** | USA | USA | USA | Belgium | Taiwan | USA |
| **Body Image** | **No** | **No** | **No** | **Yes**  (to some extent).  Asks about ‘cosmetic discomfort’ regarding ‘shape of the abdomen’ and ‘hernia site’. | **No** | **Yes**  (to some extent). Changes of the body and patient perspectives related to this are based on the operative repair itself. Not the CAWH. Equally, there is a question related to attractiveness ‘without clothes on’. From this study we know that body image is affected with or without clothing and also before repair. |
| **Mental Health** | **No** | **No** | **Yes**  (to some extent). Asks about ‘feelings’ and ‘feeling blue’ due to CAWH but does not explicitly ask about depression and anxiety. | **No** | **No** | **Yes**  (to some extent).  Asks directly about anxiety. However, depression related questions are related to how well the patient felt prepared by their surgical team for this during their recovery period, rather than how the CAWH had affected their mental health. |
| **Symptoms** | **Yes**  Physical activity based only | **Yes**  but mesh related pain only | **Yes**  Physical activity and pain. Does not ask about balance, bowel and urinary symptoms | **Yes**  Pain and restriction related to certain activities e.g. sports and heavy labour. | **Yes**  Pain, pain related to physical activity, mesh sensation, restriction of activities | **Yes**  Physical activity, pain and sleep. Does not ask about balance, bowel and urinary symptoms |
| **Interpersonal relationships including sexual relationships** | **Yes**  Sexual activity only. Does not ask about wider social relationships | **No** | **Yes**  Sexual activity only. Does not ask about wider social relationships | **No** | **Yes**  Sexual ‘discomfort’ only.  Does not ask about wider social relationships | **No** |
| **Employment** | **No** | **No** | **Yes**  ‘Accomplishing less at work’ only | **No** | **Yes**  To some extent. Asks whether the hernia ‘elicits economic burden’ | **No** |

^1^ Note that the AHQ is currently unavailable to other research groups. Therefore, this assessment is based on a form similar to the AHQ available here on the Penn Medicine website: <https://www.pennmedicine.org/for-patients-and-visitors/find-a-program-or-service/hernia-program/penn-hernia-research>
