# Supplementary material for: Health-related quality of life in abdominal wall hernia: let’s ask patients what matters to them?
Source: Hernia. 2022 Apr 12;26(3):795–808. doi: 10.1007/s10029-022-02599-6 (PMC9003180; doi:10.1007/s10029-022-02599-6)
Supplement: Supplementary file 5 — Supplementary file5 (DOCX 24 KB) [file 10029_2022_2599_MOESM5_ESM.docx]

Table 1. Summary of strategies used to ensure trustworthiness, adapted from Nobel & Smith (2015) (36)

| Strategy quoted by Nobel & Smith (2015) | How this was addressed by the study methods | Reference |
| --- | --- | --- |
| *“Accounting for personal biases which may have influenced findings”* | - Reflexive field journal and bracketing strategies - Audit decision trail - Regular discussions with supervisors PC and SC | (37) |
| *“Acknowledging biases in sampling and ongoing critical reflection of methods to ensure sufficient depth and relevance of data collection and analysis”* | - Reflexive field journal and bracketing strategies - Evidenced based interview protocol | (38) |
| *“Meticulous record keeping, demonstrating a clear decision trail and ensuring interpretations of data are consistent and transparent”* | - Reflexive field journal and bracketing strategies - Audit decision trail - Regular discussions with supervisors PC and SC - External peer review by researchers at York St. John University | (38)  (39) |
| *“Establishing a comparison case/seeking out similarities and differences across accounts to ensure different perspectives are represented”* | - Interpretive Phenomenological Analysis - Independent data review and analysis by supervisors PC and SC - Triangulation - Rich verbatim participant descriptions to support findings | (37)  (40) |
| *“Demonstrating clarity in terms of thought processes during data analysis and subsequent interpretations”* | - Reflexive field journal and bracketing strategies - Carefully constructed topic guide with use of prompts and probes - Rich verbatim participant descriptions to support findings | (38) |
| *“Engaging with other researchers to reduce research bias”* | - Peer review by research and development department at York Hospital - Discussions with supervisors - External peer review by researcher at York St. John University (MM) | (38) |
| *“Different methods and perspectives help produce a more comprehensive set of findings.”* | - Data triangulation | (38)  (39)  (41)  (42) |
